# Electrocardiogram phenotypes in hypertrophic cardiomyopathy caused by distinct mechanisms: apico-basal repolarization gradients vs. Purkinje-myocardial coupling abnormalities

**DOI:** 10.1093/europace/euy226

**Published:** 2018-11-23

**Authors:** Aurore Lyon, Alfonso Bueno-Orovio, Ernesto Zacur, Rina Ariga, Vicente Grau, Stefan Neubauer, Hugh Watkins, Blanca Rodriguez, Ana Mincholé

**Affiliations:** 1 Department of Computer Science, University of Oxford, Wolfson Building, Parks Rd, Oxford, UK; 2 Institute of Biomedical Engineering (IBME), Department of Engineering Science, University of Oxford, Oxford, UK; 3 Division of Cardiovascular Medicine, Radcliffe Department of Medicine, University of Oxford, Oxford, UK

**Keywords:** Hypertrophic cardiomyopathy, Computer modelling, Personalized simulations, Cardiac magnetic resonance imaging, Electrocardiography

## Abstract

**Aims:**

To identify key structural and electrophysiological features explaining distinct electrocardiogram (ECG) phenotypes in hypertrophic cardiomyopathy (HCM).

**Methods and results:**

Human heart–torso anatomical models were constructed from cardiac magnetic resonance (CMR) images of HCM patients, representative of ECG phenotypes identified previously. High performance computing simulations using bidomain models were conducted to dissect key features explaining the ECG phenotypes with increased HCM Risk-SCD scores, namely Group 1A, characterized by normal QRS but inverted T waves laterally and coexistence of apical and septal hypertrophy; and Group 3 with marked QRS abnormalities (deep and wide S waves laterally) and septal hypertrophy. Hypertrophic cardiomyopathy abnormalities characterized from CMR, such as hypertrophy, tissue microstructure alterations, abnormal conduction system, and ionic remodelling, were selectively included to assess their influence on ECG morphology. Electrocardiogram abnormalities could not be explained by increased wall thickness nor by local conduction abnormalities associated with fibre disarray or fibrosis. Inverted T wave with normal QRS (Group 1A) was obtained with increased apico-basal repolarization gradient caused by ionic remodelling in septum and apex. Lateral QRS abnormalities (Group 3) were only recovered with abnormal Purkinje-myocardium coupling.

**Conclusion:**

Two ECG-based HCM phenotypes are explained by distinct mechanisms: ionic remodelling and action potential prolongation in hypertrophied apical and septal areas lead to T wave inversion with normal QRS complexes, whereas abnormal Purkinje-myocardial coupling causes abnormal QRS morphology in V4–V6. These findings have potential implications for patients’ management as they point towards different arrhythmia mechanisms in different phenotypes.

**What’s new?**

- Simulations using multiscale whole-organ computer models based on cardiac magnetic resonance images dissect the influence of structural and electrophysiological abnormalities on the electrocardiogram (ECG) in hypertrophic cardiomyopathy (HCM).
- Increased wall thickness, fibre disarray, bundle block, or myocardial abnormalities do not account for the different ECG phenotypes identified in HCM.
- An abnormal Purkinje-myocardium coupling is the only mechanism we found that could explain the QRS abnormalities with deep and wide S waves in lateral leads observed in one patient group.
- Apico-basal repolarization heterogeneities due to ionic remodelling in coexisting regions of septal and apical hypertrophy explain the normal QRS and inverted T waves observed in the phenotype associated with increased sudden cardiac death risk score.

## Introduction

Hypertrophic cardiomyopathy (HCM) has remained a challenge due to the extreme heterogeneity in its clinical course. Although most patients are asymptomatic, HCM is a major cause of sudden cardiac death (SCD) in young people due to ventricular arrhythmias. These life-threatening arrhythmias are effectively aborted by implantable cardioverter-defibrillators (ICDs) and the focus of risk stratification is to target high-risk patients with an ICD. The electrocardiogram (ECG) is abnormal in the majority of patients from abnormal Q waves, ST segments, T-waves to wide QRS.^1^^,^^2^ However, the electrocardiographic signature of HCM has been inconclusive for reliable risk stratification.^3^

Progress towards risk stratification was achieved in recent work,^4^ where four HCM phenotypes were identified using computational analysis of the ECG. The greatest deviation from a normal ECG occurred in two of the four ECG phenotypes: Group 1A and Group 3.^5^ Patients in Group 1A had normal QRS morphology, inverted T waves laterally, increased HCM Risk-SCD scores, and coexisting septal and apical hypertrophy. Patients in Group 3 had QRS abnormalities with deep wide S waves laterally and septal hypertrophy. The structural and electrophysiological sources of these different ECG phenotypes in HCM remain unclear and complex, as they may include hypertrophy, fibre disarray, fibrosis, ionic remodelling, as well as Purkinje abnormalities.^6–8^ A better understanding of the electrophysiological and structural basis of the HCM phenotypes may help to improve personalized patient management, as the mechanisms underlying arrhythmic risk may differ.

The goal of this study is to identify the key HCM structural and electrophysiological features underlying the patients’ phenotypes with more abnormal ECGs. We hypothesise that abnormalities in the microstructure such as fibre disarray may explain abnormal electrical activation and marked QRS abnormalities in Group 3, and that apical HCM ionic remodelling may lead to primary repolarization prolongation and inverted T waves in Group 1A. To achieve these aims, we exploit the power of high performance computing simulations of the ECG using human biophysically-detailed torso-ventricular models personalized to the patients’ cardiac magnetic resonance (CMR) images.^9^

## Methods

### Hypertrophic cardiomyopathy phenotypes

**Figure 1:**
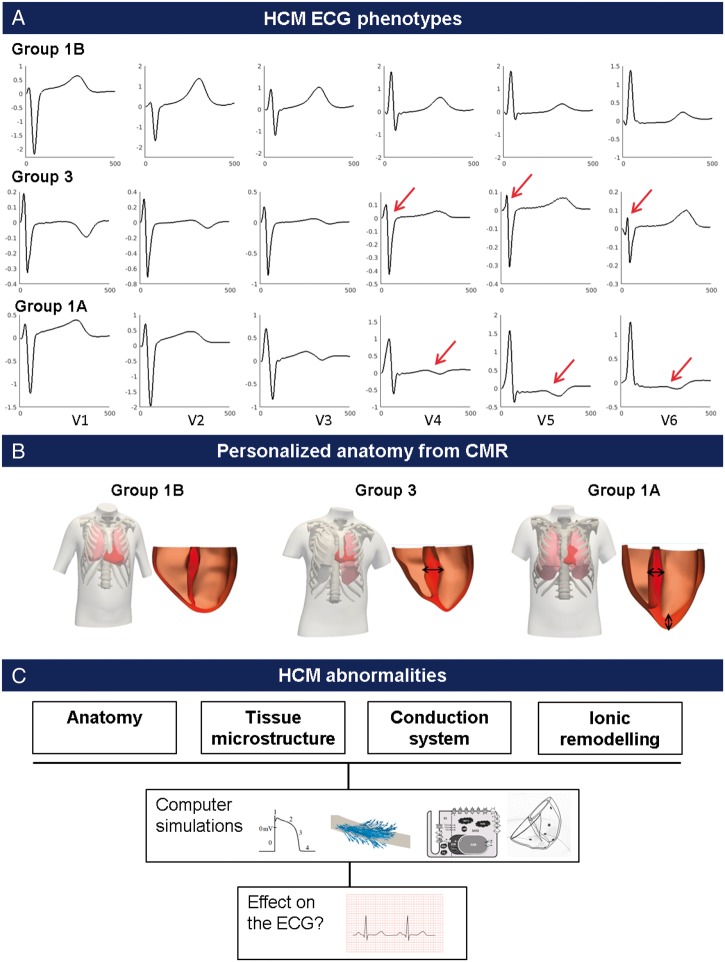
Investigation of the effect of HCM abnormalities on the ECG. (*A*) ECG morphologies of the HCM phenotypes studied: Group 1B with normal ECG morphology in V4–V6, Group 3 with QRS abnormalities with deep and wide S waves in V4–V6, and Group 1A with normal QRS morphology but inverted T waves in V4–V6. (*B*) Personalized 3D volumetric meshes of the heart and torso are computed for each representative patient. (*C*) The effect of HCM abnormalities (anatomy, tissue microstructure, conduction system, and ionic remodelling) on the ECG are investigated using computer simulations. CMR, cardiac magnetic resonance; ECG, electrocardiogram; HCM, hypertrophic cardiomyopathy.

This study focused on the interpretation of the ECG morphologies of patients from Group 1A and 3 identified in Ref.,^4^ which exhibited the greatest deviation from a normal ECG. As a reference, simulations were also conducted for Group 1B, which had normal QRS and T wave morphologies. One representative patient was chosen from each of the HCM phenotypic groups based on the presence of the characteristic ECG features (Group 1B: normal QRS, upright T waves in V4–V6, Group 1A: normal QRS, inverted T waves in V4–V6, Group 3: short R duration and amplitude, long S duration and amplitude in V4–V6) (*Figure 1A*), and the presence of the characteristic hypertrophic areas on the CMR images (Group 1A: mixed pattern of septal and apical hypertrophy, Groups 1B and 3: septal hypertrophy) (*Figure 1B*).

### Cardiac magnetic resonance-based heart–torso models

Biventricular heart, torso, lungs, and ribs meshes were derived from CMR scans of each patient by image processing, and virtual ECG electrodes were positioned on the torso surface in standard placement to simulate the 12-lead ECG (see Supplementary material online, 1.1).

Human ventricular membrane kinetics were represented by modified versions of the human ventricular electrophysiological O’Hara–Rudy model for healthy^10^ and HCM cardiomyocytes.^7^ The models are biophysically-detailed and include representation of sodium, calcium, and potassium ionic currents as well as calcium dynamics. Hypertrophic cardiomyopathy-related ionic remodelling was represented by altering sodium, calcium, and potassium currents as described in Ref.^7^ Myocardial and torso conductivities and ventricular fibre architecture were set as described in Supplementary material online, 1.2. Electrophysiological heterogeneities were introduced to represent the dispersion of action potential duration (APD) in ventricular repolarization (see Supplementary material online, 1.3). To simulate sinus rhythm, endocardial activation was initiated in root nodes (or sites of earliest activation) connected with a fast activating endocardial layer as in Ref.,^9^ representing a phenomenological model of Purkinje-ventricular coupling connected to the rest of the working myocardium (see Supplementary material online, 1.2). The location of early activation nodes in all our heart geometries were selected to follow correspondent anatomical locations by mapping homologous anatomical regions, such as insertion points, apex, and lateral walls, among all geometries. The electrophysiological model was therefore kept the same in all cases in order to isolate the effect of each HCM-related feature on the ECG, rather than aiming at matching the clinical ECG in a patient-specific approach. Variability in body conductivities, which has been shown to strongly affect QRS amplitude^9^ may be responsible for differences in wave amplitude between simulated and clinical ECGs. Analysis and comparisons of the ECG signals were therefore made in terms of overall morphology and polarity.

### Hypertrophic cardiomyopathy abnormalities

Several hypotheses were tested to explain the ECG phenotypes in HCM, based on CMR-based features and HCM electrophysiological and structural understanding (*Figure 1C*). The anatomy (increased wall thickness) was intrinsically included in the heart–torso models as they were personalized from CMR images. Abnormalities in tissue microstructure were also investigated. Fibre disarray was modelled as an isotropic conduction in the regions of low fractional anisotropy obtained from diffusion tensor imaging (DTI) data. Decreased conduction velocity (such as due to fibrosis, known to affect HCM hearts^6^) was modelled in those areas as a four-time reduction in tissue conductivities, resulting in reduced conduction velocity of 20 cm/s. Conversely, hypertrophied cells have been shown to lead to increased conduction velocity in earlier stages of left ventricular hypertrophy.^11^ Such an effect was therefore additionally investigated by a four-time increase in conductivities, leading to an increased conduction velocity in the regions of hypertrophy.

Bundle branch blocks have also been reported in HCM,^12^^,^^13^ and the effect of various blocks in the conduction system on the ECG were therefore tested (left bundle branch block, LBBB; left ventricular posterior and anterior hemiblocks; right bundle branch block, RBBB) by deleting associated root nodes in the model.^9^ In addition, slurred S wave morphologies in V5–V6 may be typical of RBBB, suggesting its potential implication in the QRS abnormalities of Group 3 patients. Abnormal Purkinje-myocardium coupling, as reported in HCM patients,^8^ was induced by a mismatch between the fast endocardial layer and the myocardium introduced by doubling conductivities. Fast propagation regions have been shown capable of creating unidirectional propagation block because of source-sink mismatch.^14^ Finally, HCM ionic remodelling was introduced in the regions of hypertrophy, as described in Ref.^7^ This consisted in an alteration of the cellular ionic properties, mainly including an increase of the late sodium and the L-type calcium current, reduction of the potassium currents, and remodelling of the calcium handling subsystem.

## Results

### Effect of cardiac anatomy on the electrocardiogram phenotypes in hypertrophic cardiomyopathy

**Figure 2:**
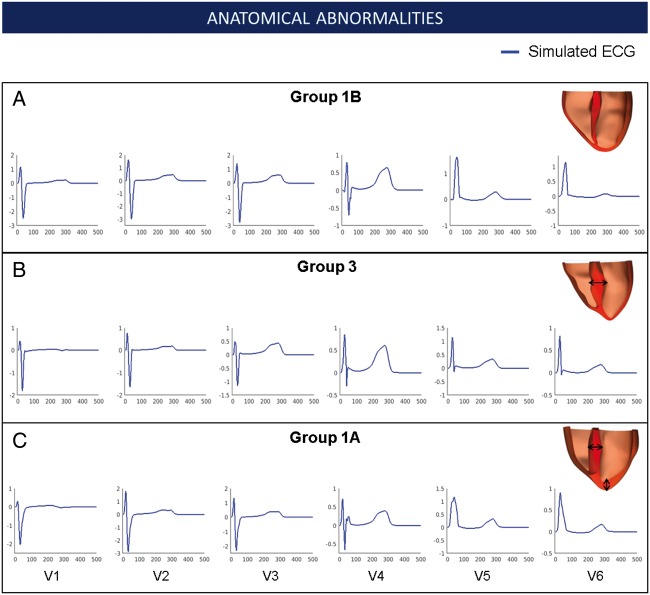
Effect of the anatomy on the ECG phenotypes observed in HCM. Simulated ECG of representative patients in Group 1B (*A*), Group 3 (*B*), and Group 1A (*C*) for leads V1–V6 along with their personalized heart anatomy. Arrows indicate areas of hypertrophy. ECG, electrocardiogram; HCM, hypertrophic cardiomyopathy.

The anatomical effects of cardiac shape, hypertrophy distribution, and extent on the ECG were investigated by patient-specific geometries derived from CMR images (*Figure 2*). Despite characteristic distributions of hypertrophy in each group, the simulations yielded similar QRS morphology and upright T waves in all groups, which were consistent with the clinical ECG from Group 1B. As a sensitivity analysis, we also analysed the influence of varying both the intracellular and extracellular conductivities on the shape of the QRS. Conductivity changes did not lead to morphological abnormalities such as narrow tall R waves and deep wide S waves as seen in Group 3 (see Supplementary materials online, 2.1). Therefore, increased wall thickness alone did not explain abnormalities in QRS morphology in Group 3, or the T wave inversion in Group 1A.

### Effect of abnormalities in tissue microstructure on the QRS complex

#### Effect of local isotropic electrical propagation due to fibre disarray

Hypertrophic cardiomyopathy hearts exhibit structural abnormalities such as fibre disarray, which corresponds to a disarrangement of the cardiac fibres and a loss of the normal electrical anisotropy property of the cardiomyocytes. This may affect ventricular activation, and in turn the QRS complex, as observed in Group 3 patients. However, the introduction of isotropic electrical conduction in areas with low fractional anisotropy from DTI made little change to the simulated ECG in Group 3 (see Supplementary material online, 2.2).

#### Effect of altered conduction velocity by fibrosis and hypertrophy

**Figure 3:**
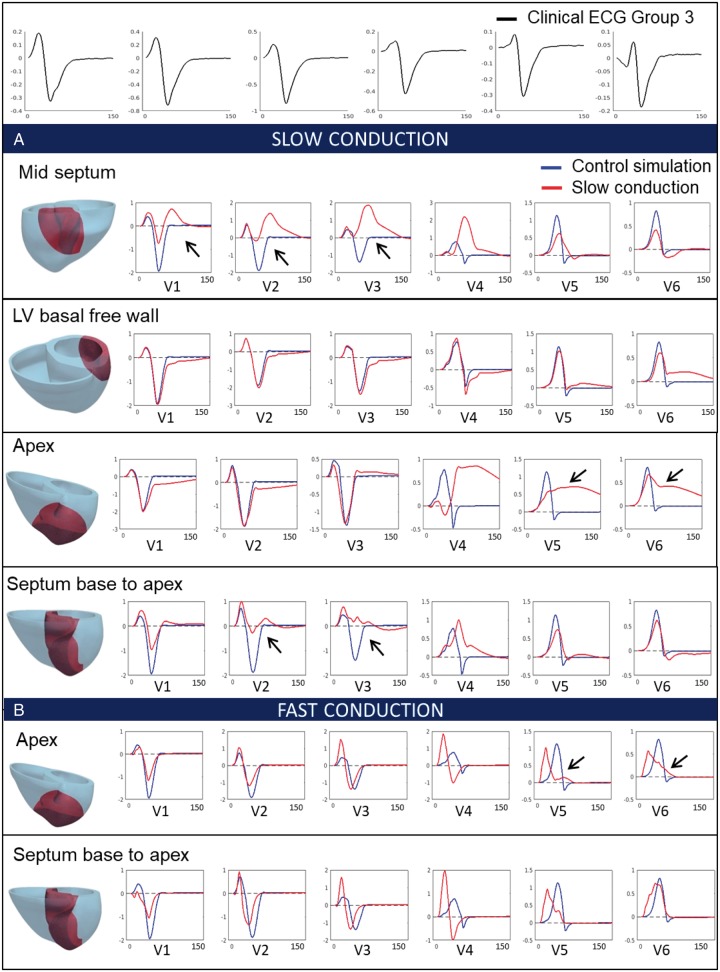
Effect of changes in conduction speed (due to fibrosis, hypertrophy) on the QRS. (Top) Clinical ECG of Group 3 patient (black). (*A*) Simulated QRS with slow conduction in various locations of the myocardium (mid-septum, left ventricular basal free wall, apex and septum from base to apex), highlighted in red. (*B*) Simulated QRS with fast conduction in various locations of the myocardium (mid-septum, left ventricular basal free wall, apex and septum from base to apex), highlighted in red. Simulated QRS in control conditions is represented in blue. Black arrows represent lead of interest discussed in the text. ECG, electrocardiogram; LV, left ventricular.

We then evaluated the potential impact of slowed electrical conduction in four different regions of the myocardium on the ECG (*Figure 3A*). Slowing conduction in a mid-septal region yielded abnormal QRS morphologies in septal and anterior leads V1–V4. Slowing conduction in the left ventricular basal free wall did not affect the QRS in the septal leads V1–V3, despite a smoother upright S slope to the baseline. QRS in leads V4–V6 showed a similar morphology but increased width. Slowing conduction in the apex affected mostly leads V4–V6 with severely abnormal patterns of wide QRS. Finally, slowing down conduction in a region covering the entire septum from base to apex led to severe QRS abnormalities in septal leads V2–V4, with reduced QRS amplitude and increased width, but similar morphologies in leads V5–V6. The abnormalities in leads V1–V4 with the entire septum affected were milder than in the case of the mid septal region being affected. This was explained by the large activation delays in the anterior of the ventricles caused by the region of slow conduction in the mid septum, which extended to the anterior. These were not present in the entire septum case (see activation maps in Supplementary material online, 2.3, *Figure S4*). Importantly none of the changes introduced resulted in the deep S waves in leads V4–V6 observed in Group 3.

We then considered that the increased surface-to-volume ratio of hypertrophic myocytes could also lead to an increase in electrical propagation speed.^11^ Faster conduction in both apical and septal regions increased the QRS amplitude and reduced the QRS width in leads V3–V4, but did not lead to a deep S wave in leads V5 and V6 (*Figure 3B*). Altogether, these results showed that positive or negative changes in the conduction speed in local regions of the myocardium did not produce a deep S wave in leads V4–V6, and therefore could not explain the abnormalities observed in Group 3 patients.

### Effect of abnormalities in the conduction system on the QRS complex

#### Effect of altered early activation sites

**Figure 4:**
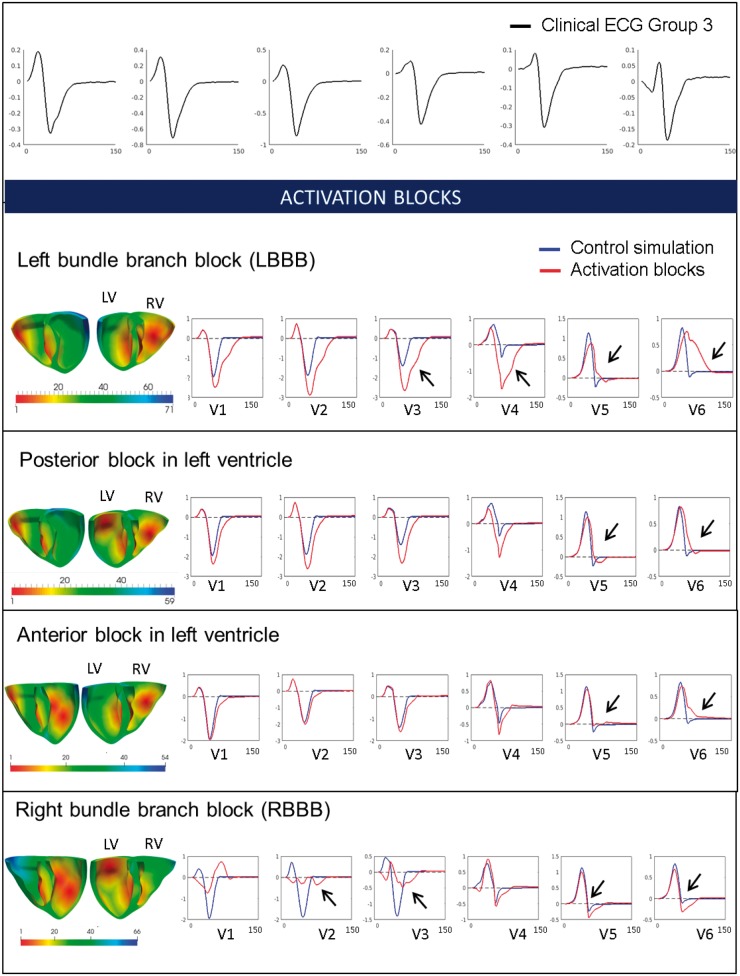
Effect of early activation sites knock-outs on the QRS. (Top) Clinical ECG of Group 3 patient (black). Changes in the activation sequence with different blocking combinations (left bundle branch block, posterior block, anterior block, and right bundle branch block). Activation maps in both ventricles are shown along with the specific simulated ECG (red), normal simulation (blue) in leads V1–V6. Black arrows represent lead of interest discussed in the text. ECG, electrocardiogram.

Presence of branch blocks, such as LBBB or RBBB, has been reported in HCM.^12^^,^^13^ We, therefore, investigated the effect on the ECG of hypertrophic ventricles of various combinations of activation blocks (*Figure 4*). Simulation results showed that various combinations of activation blocks in the left ventricle affected the duration of the activation, and therefore, the width of QRS complexes, but did not change the overall morphology of the QRS in lateral leads. Blocking the activation in the left ventricle free wall (LBBB) led to wider QRS complexes in V1–V6 due to slow activation of the left ventricle. Knocking out the posterior branch of the left ventricle did not affect the morphology of the QRS in any leads, slightly increasing the amplitude of the QRS in V2–V4. Knocking out the anterior branch of the left ventricle had very little effect on the QRS, slightly increasing the QRS width, and did not affect wave polarities. Finally, we investigated the effect of blocking the early activation in the right ventricle (RBBB). This led to a slight increase in amplitude and width of the S waves in lateral leads, but maintained the prominence of the R wave in V4–V6. The precordial leads V1–V3 were strongly affected and showed abnormalities not observed on the ECG of Group 3 patients.

We concluded that altering the activation sequence of the left and right ventricles did not lead to the morphological abnormalities observed in the lateral leads V4–V6 combined with normal QRS morphology in leads V1–V3.

#### Effect of abnormal Purkinje-myocardium coupling

**Figure 5:**
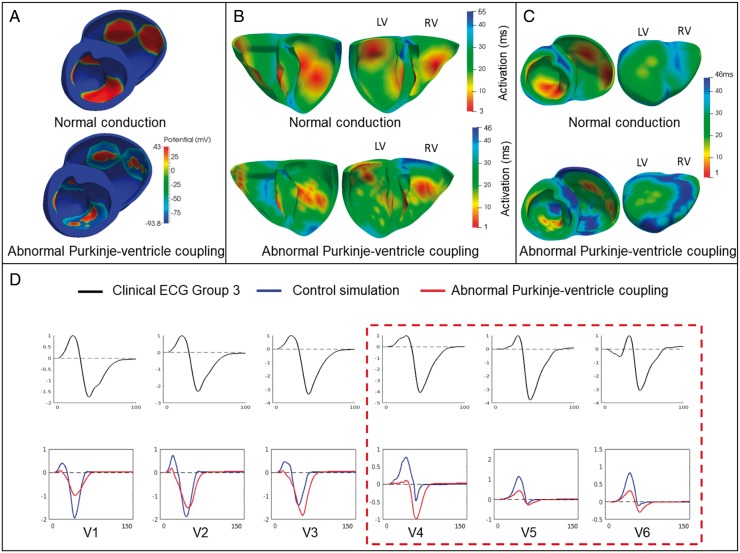
Effect of an abnormal Purkinje-myocardium coupling on the QRS. (*A*) Patchy activation in the simulation with abnormal Purkinje-myocardium coupling compared with the normal conduction. (*B*) Activation maps for the simulation with abnormal Purkinje-myocardium coupling show diffuse activation. (*C*) The simulation with abnormal Purkinje-myocardium coupling shows regions of late activation (blue) compared with the normal simulation. (*D*) Modelling of altered fast propagation system (red) with normal simulation (blue) in leads V1–V6. Clinical ECG is shown in black. ECG, electrocardiogram.

The abnormal Purkinje-myocardium coupling, modelled as described in Methods section, led to a patchy activation and a diffuse activation sequence compared with the control simulation (*Figure 5A* and *B*). This led to the presence of late-activated areas in the myocardium compared with the control simulation, especially in the right ventricle base and endocardium, and the apex (*Figure 5C*). This translated into deep and wide S waves in lateral leads V4–V6 of the ECG, without affecting the morphology of the septal leads (*Figure 5D*). Therefore, an abnormal coupling between branches of the Purkinje system and the ventricular endocardium was the only way to reproduce the QRS polarity abnormalities in Group 3, with abnormal deep and wide S wave in the lateral leads. Affecting the myocardial propagation or the early activation sequence did not explain them.

### Effect of hypertrophic cardiomyopathy ionic remodelling on T wave polarities

**Figure 6:**
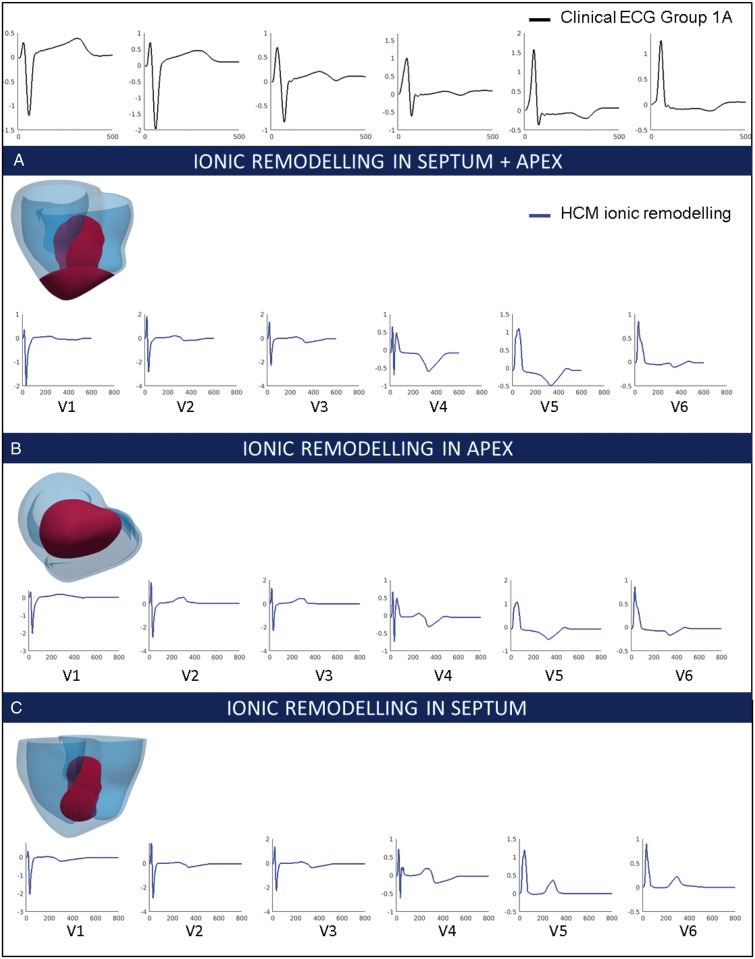
Effect of ionic remodelling in the region of hypertrophy on the T wave. (Top) Clinical ECG of Group 1A patient (black). (*A*) Simulated ECG with ionic remodelling in the septum and apex (red area) for leads V1–V6. (*B*) Simulated ECG with ionic remodelling in the apex (red area) for leads V1–V6. (*C*) Simulated ECG with ionic remodelling in the septum (red area) for leads V1–V6. ECG, electrocardiogram; HCM, hypertrophic cardiomyopathy.

In order to investigate the phenotype of Group 1A patients, we hypothesized that HCM ionic remodelling, resulting in longer APD in the region of hypertrophy (combined septum and apex) may translate in an inversion of the T wave in leads V4–V6. The HCM ionic remodelling described in Ref.^7^ was modelled in the septum and apex, in agreement with the mixed pattern of hypertrophy of these patients. This led to an increased APD in the region of hypertrophy (455 ms with HCM ionic remodelling vs. 324 ms without). The simulated T waves showed inversions in the lateral leads V4–V6, while the T waves in other leads remained unaffected (*Figure 6A*). This replicated the morphology of the clinical ECG of Group 1A patients in all leads. The QRS complex, representing the activation phase of the cardiac cycle, was not affected by HCM ionic remodelling. The ionic remodelling modelled in the apex only also led to inverted T waves in the lateral leads (*Figure 6B*). Interestingly, ionic remodelling modelled only in the septum (*Figure 6C*) did not yield inverted T waves in leads V4–V6, confirming the crucial role of the apical hypertrophy in Group 1A phenotype. Indeed, Group 1A was the only one exhibiting mixed apico-basal hypertrophy and inverted T waves, while the other groups only exhibited septal hypertrophy. This confirmed that apico-basal heterogeneities due to ionic remodelling in the septal and apical hypertrophied region may be responsible for the primary repolarization abnormalities observed in Group 1A, expressed as inverted T waves and normal QRS morphology.

## Discussion

High performance computing simulations using CMR-informed human torso-ventricular models have provided strong evidence that the two phenotypes displaying more abnormal ECGs in HCM may be explained by two distinct mechanisms, with potential relevance to patients’ management. Hypertrophic cardiomyopathy patients with inverted T wave and normal QRS (Group 1A) were affected by apico-basal repolarization gradients caused by ionic remodelling in septal and apical hypertrophy regions. Hypertrophic cardiomyopathy patients with QRS abnormalities in the lateral leads (Group 3) were affected by abnormal coupling between Purkinje branches and myocardium. These simulations have also shown that anatomical and microstructural abnormalities alone could not explain the distinct ECG abnormalities identified in each HCM phenotypes.

### Hypertrophic cardiomyopathy structural abnormalities: hypertrophy, disarray and fibrosis

Ventricular anatomical abnormalities alone did not yield any of the ECG abnormalities identified in the HCM phenotypes, either QRS abnormalities or T wave inversions. Therefore, this suggests that left ventricular hypertrophy may not be a major driver in the identification of the distinct ECG phenotypes in HCM and may not, on its own, strongly contribute towards higher arrhythmic risk in HCM patients, as highlighted in previous studies.^15^ The effects of other HCM abnormalities, such as fibre disarray (modelled as isotropic electrical propagation) or changes in conduction speed due to fibrosis or hypertrophy, were simulated. However, none of these changes in myocardial conduction reproduced the QRS abnormalities observed in Group 3. This agrees with studies of HCM families exhibiting myofibre disarray in the absence of ventricular hypertrophy, where no QRS abnormalities were reported.^16^ Altering the activation sequence by modelling various activation knock-outs did not yield these abnormal morphologies either.

### Abnormal Purkinje-myocardium coupling

Affecting the Purkinje-myocardium coupling was the only way to reproduce the abnormalities in QRS complex characteristic of Group 3. We modelled a reduced coupling in the Purkinje branches coupled to the endocardium, which led to a patchy activation with areas of late activation, and translated to deep and wide S waves in lateral leads V4–V6 on the ECG. This is in agreement with a study reporting large Purkinje-muscle junction delays in HCM patients.^8^ These findings may illustrate an abnormal Purkinje conduction in these patients, also in agreement with studies reporting 23% of HCM patients with abnormal His-Purkinje conduction system.^17^ Cases of HCM patients with abnormal His-Purkinje system were also reported in Ref.,^18^ with the mention of a patient exhibiting a deep S wave morphology in V6. Invasive studies such as endocardial mapping may help investigate the extent of this possible mechanism. Future studies based on late gadolinium enhancement data, exploring the presence of myocardial fibrosis or scaring affecting the coupling of Purkinje fibres with the endocardium,^19^ may also provide insight.

### Apex-base repolarization heterogeneities

Longer APD in the hypertrophic area (combined septum and apex) due to HCM ionic remodelling led to T wave inversions in leads V4–V6 in Group 1A, without affecting the normal morphology of the QRS complex. The location of these ionic abnormalities was in agreement with the unique hypertrophy distribution in these patients. Group 1A was indeed the only HCM phenotype exhibiting coexistence of apical and septal hypertrophy, suggesting that ionic remodelling abnormalities may occur in the hypertrophied region of these patients.

### Clinical implications

These results highlighted two different potential mechanisms for ECG abnormalities associated with increased risk of SCD in HCM. Abnormal Purkinje conduction may explain the QRS abnormalities in the lateral leads in Group 3 patients with deep and wide S waves, while ionic remodelling in the region of hypertrophy may be the mechanism responsible for inverted T waves in Group 1A patients. Group 1A patients presented a higher HCM risk score of SCD compared to other groups, especially Group 3.^4^ This suggests that repolarization gradients due to ionic remodelling may play a key role in arrhythmogenesis, as suggested in Ref.^20^ Conduction abnormalities in the Purkinje system may be less pro-arrhythmic as they may not perturb the overall synchronicity of the cardiac cycle. The very different nature of these two mechanisms shows potential implications in personalised treatment of HCM patients. For example, patients with Group 1A phenotype may benefit from pharmacological block of late sodium current as suggested in Ref.,^7^ whereas this may be ineffective in Group 3 patients. Our study reinforces the importance of computational ECG phenotyping and modelling in risk assessment and patient management. Future work using techniques, such as endocardial or epicardial mapping, will be needed to verify the hypotheses generated and improve the understanding of the connection between structure and function in HCM patients.

## Conclusions

High performance computing simulations using CMR-informed torso-ventricular models were conducted to unravel key structural and electrophysiological features explaining abnormalities in specific ECG-based phenotypes of HCM patients.^4^ A personalized whole-body simulation framework allowed the impact of hypertrophy, fibre disarray, conduction speed and ionic remodelling on the ECG to be quantified. Simulations highlight the existence of distinct mechanisms for the phenotypes at higher risk. An abnormal Purkinje-myocardium coupling may explain the QRS abnormalities in one of the HCM phenotypes at higher risk, whereas apico-basal repolarization gradients in the regions of hypertrophy may explain the primary repolarization abnormalities observed in another phenotype with increased HCM Risk-SCD scores, exhibiting inverted T wave and normal QRS with coexistence of septal and apical hypertrophy. Overall, this study provided novel hypotheses for structure-function relationships in HCM that may be tested by further studies. We confirm the importance of HCM phenotyping for patient management, and the potential of computer modelling and simulation in describing and understanding cardiac heterogeneity and disease.

## Supplementary Material

Supplementary Data
