# Supplementary material for: Electrocardiogram phenotypes in hypertrophic cardiomyopathy caused by distinct mechanisms: apico-basal repolarization gradients vs. Purkinje-myocardial coupling abnormalities
Source: Europace. 2018 Nov 23;20(Suppl 3):iii102–12. doi: 10.1093/europace/euy226 (PMC6251182; doi:10.1093/europace/euy226)
Supplement: Supplementary Data [file euy226_supplementary_material.pdf]

Supplementary Material

1. Methods

1.1 Anatomical mesh construction

The first step of the computational pipeline consisted in constructing personalized 3D bi-ventricular surfaces of the heart, torso, lungs and ribs from CMR images. MRI slices of the heart (long and short axis) were acquired, and pre-processing steps such as alignment (1), and manual contour delineation were applied to generate 3D surfaces of the bi-ventricular geometry from the patient's CMR images. An iterative process consisting in remeshing, smoothing, and fitting of a surface towards the input contours is performed to generate the heart surfaces following (2). Torso surface geometries were derived from combining manual contouring with a statistical model of the torso. Ribs and lungs were personalized from a template to the patient's anatomy (3).

The next step was to generate tetrahedral volumetric meshes from these surfaces using the software Tetgen (4). Each tetrahedron was defined by four nodes and allowed the propagation of the electrical signal throughout the mesh. The meshing process required the definition of a tetrahedral edge length and multiple optimization parameters. In our case, a fine and detailed mesh was required in the heart in order to obtain numerical convergence in the simulations of electrical propagation with the Chaste software. Torso, lungs and ribs were designed as passive tissues with different fixed conductivities, allowing a coarser mesh to be defined in these regions (following (5)'s work). The edge length of each element was defined as 0.4mm for the heart, 3mm for the lungs, 2mm for the ribs, and 10mm for the torso based on (5) and (6)'s benchmarking work. An adaptive meshing, providing smooth transitions between different edge lengths, was used by controlling the volume of the tetrahedra in order to ensure a smooth propagation of the electrical signal in the whole domain. In order to simulate the 12-lead ECG,

ten nodes were defined to model electrodes on the torso surface, adapting the location defined in (5) to each personalized torso by geometrical deformation.

1.2 Electrophysiological and activation model

The 3D biventricular volumetric meshes were therefore composed of a set of nodes (vertices of the tetrahedra, defined by their coordinates) and elements (tetrahedra, defined by four nodes). The electrophysiological simulation described below followed the framework published in (5). The modified version of the electrophysiological O'Hara-Rudy model of ventricular cells (7) (8) was used to define cellular activity. Intracellular conductivities in the active tissues were chosen in order to generate conduction velocities close to 67, 30 and 17 cm/s in the longitudinal, transversal and transmural directions (9). Extracellular conductivities were defined according to resistivity ratios (10). Extracellular and intracellular conductivities were adapted from (5) based on a convergence analysis. A scaling factor of 1.8 led to longitudinal and transverse conduction velocities within experimental ranges (67 and 30 cm/s). Initial conductivities were therefore set to (2.7, 0.81, 0.405 mS/cm) for intracellular conductivities and (9.81, 3.65 and 3.65 mS/cm) for extracellular conductivities. In passive tissues, isotropic conductivities were chosen to be 0.389 mS/cm in the lungs, 0.2 mS/cm in the bones, and 2.16 mS/cm in the rest of the torso (11) (12) (5). The activation sequence was then modelled by defining seven stimuli points (root nodes) in the endocardium (four in the left ventricle and three in the right ventricle) to initiate the activation. These endocardial sites of earliest activation modelled the role played by Purkinje branches in real physiology. They were adapted from (5) by computing a mapping to each patient's anatomy. The stimulus in these points was initially defined by a duration of 1ms, a magnitude of 120,000 mA/cm3, and a speed of 0.179 cm/s. The shortest distances between each node were computed using the Dijkstra algorithm (13) in the left and right ventricles, in order to model the Purkinje network and define the propagation of the stimuli from the root nodes to the rest of the endocardial surface nodes.

In order to simulate the ECG on the torso surface, ten nodes were defined to model electrodes placed on the torso, adapting the location defined in (5) to each personalized torso by geometrical deformation. Fibre orientations were computed for each geometry following a rule-based method replicating findings in (14).

1.3 Action potential duration heterogeneities

Several configurations of heterogeneities representing the dispersion of action potential duration were considered. Apex-to-base heterogeneities were modelled by introducing a gradient of I_{Ks} conductance from base to apex to reach an APD difference of 50ms. Similarly, interventricular heterogeneities were modelled by modifying the gradient of g_{Ks} between left and right ventricles (25ms APD RV to LV difference). Finally, transmural heterogeneities were defined using layers of endocardial, midmyocardial and epicardial cells with different APD properties as described in the O'Hara-Rudy model (7). The combination of apex-to-base and transmural heterogeneities with 45% endocardial cells, 25% mid-myocardial cells and 30% epicardial cells yielded the best simulations compared to the clinical ECGs and was therefore chosen in the rest of the paper.

Simulations were run using Chaste (15) on the Archer UK national supercomputer using parallelization on 960 cores.

2. **Results**

2.1 Increased wall thickness alone does not account for the ECG phenotypes

Figure S1 below compares the activation maps for patients in Group 1B, 1A and 3 in the normal configuration. The activation sequences were similar in the three hearts: the activation was triggered in the seven activation points defined, propagated through the ventricles and finished in the apex. These similar activation patterns confirmed that the various types of abnormalities observed on the ECG phenotypes may be due to other factors than the anatomy alone.

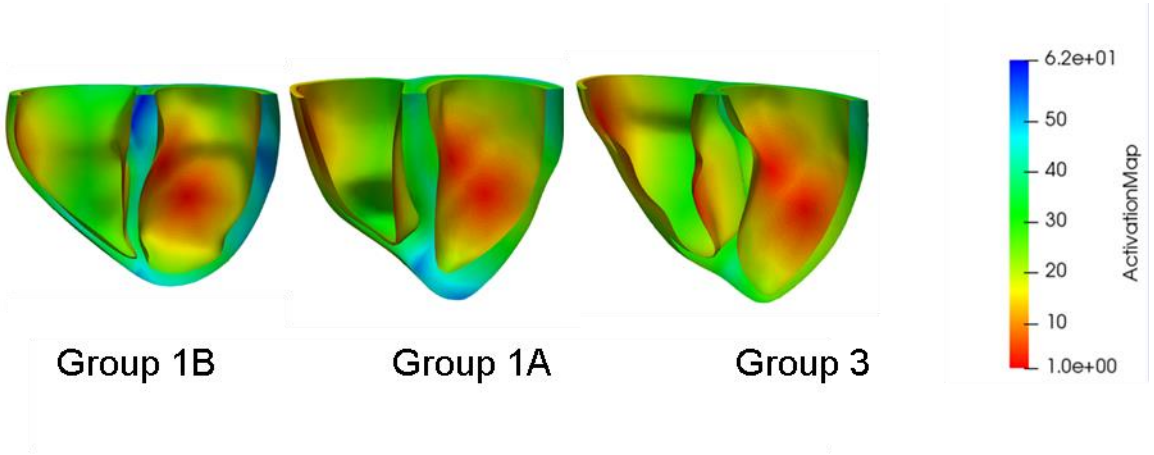

Figure S1: Activation maps (ms) for patients in Group 1B, 1A and 3. Activation is initiated in the seven activation nodes and propagates in the ventricles to the apex.

As a sensitivity analysis, we analysed the influence of varying both the intracellular and extracellular conductivities on the shape of the QRS, to explore whether these changes could explain the QRS abnormalities observed in Group C patients. Results are shown on Figure S2 below. Reducing the conductivities increased the amplitude and width of the QRS complexes, especially the R wave in V1-3, but did not change the morphology and polarity of the QRS waveforms, especially in leads V5-6. Doubling the conductivities did not yield severe changes in QRS morphology. It slightly reduced the width of the QRS in all leads, overall decreased the amplitude of the QRS (especially the R wave in leads V1-4), and slightly increased the amplitude of the S wave in V5-6 while still exhibiting a tall R wave in V5-6. In all cases, the

QRS in V4-6 still exhibited a large R amplitude with small S wave, different from the real ECG showing a small R amplitude with deep and wide S wave. These results suggest that simple homogeneous conductivity changes do not lead to severe morphological abnormalities in the lateral leads of Group 3 patients.

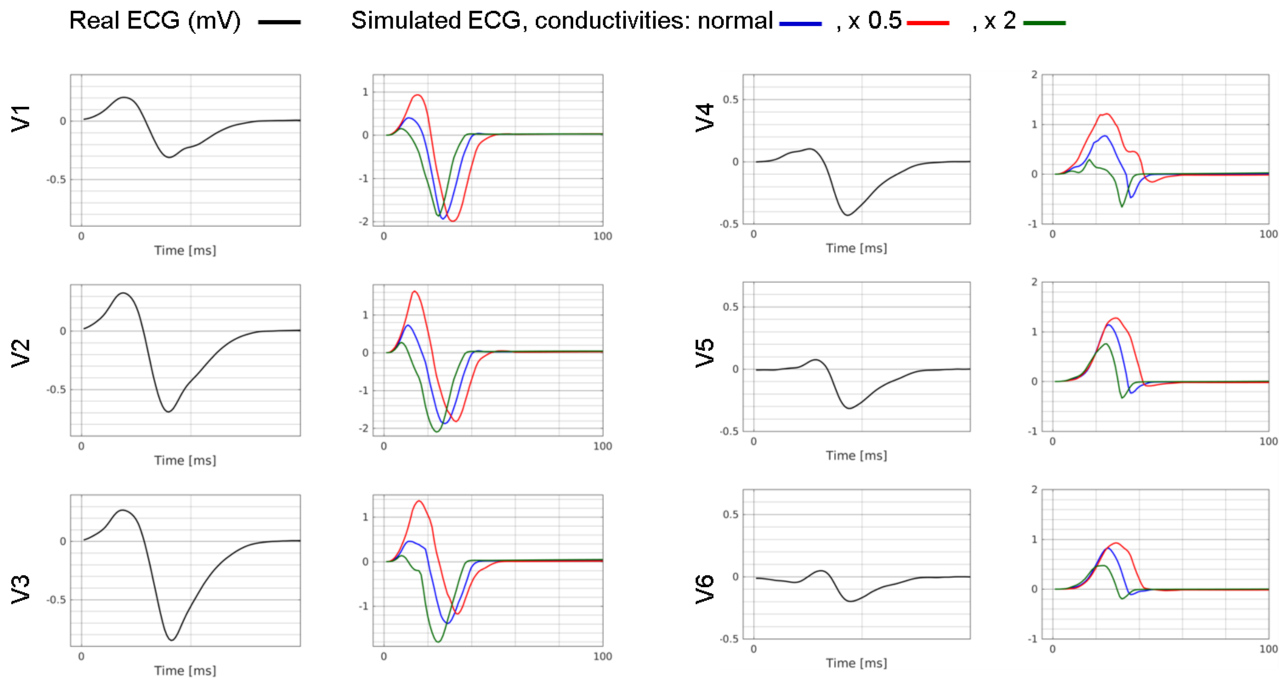

Figure S2: Analysis of conductivity changes on the ECG. Real ECG (black) is represented along with a normal simulation (blue), a simulation with intracellular and extracellular conductivities divided by 2 (red), and a simulation with intracellular and extracellular conductivities multiplied by 2 (green).

2.2 Fibre disarray modelled as isotropic propagation

Diffusion tensor imaging (DTI) provides information on the regions that may be affected by fibre disarray. Low fractional anisotropy (FA) may be the indicator of disarray, fibrosis, cell enlargement, or a combination of all. Low FA was observed in the basal and mid slices, in the anterior RV/LV insertion point at the epicardial border (Figure S3A). Patchy low FA was also

noticeable all around the septum. In the apical slice, low FA was seen in the endocardium of the anterior and posterior LV/RV insertion point. As observed in Figure S3B, isotropic propagation hardly affected the QRS complex in leads V1-V6.

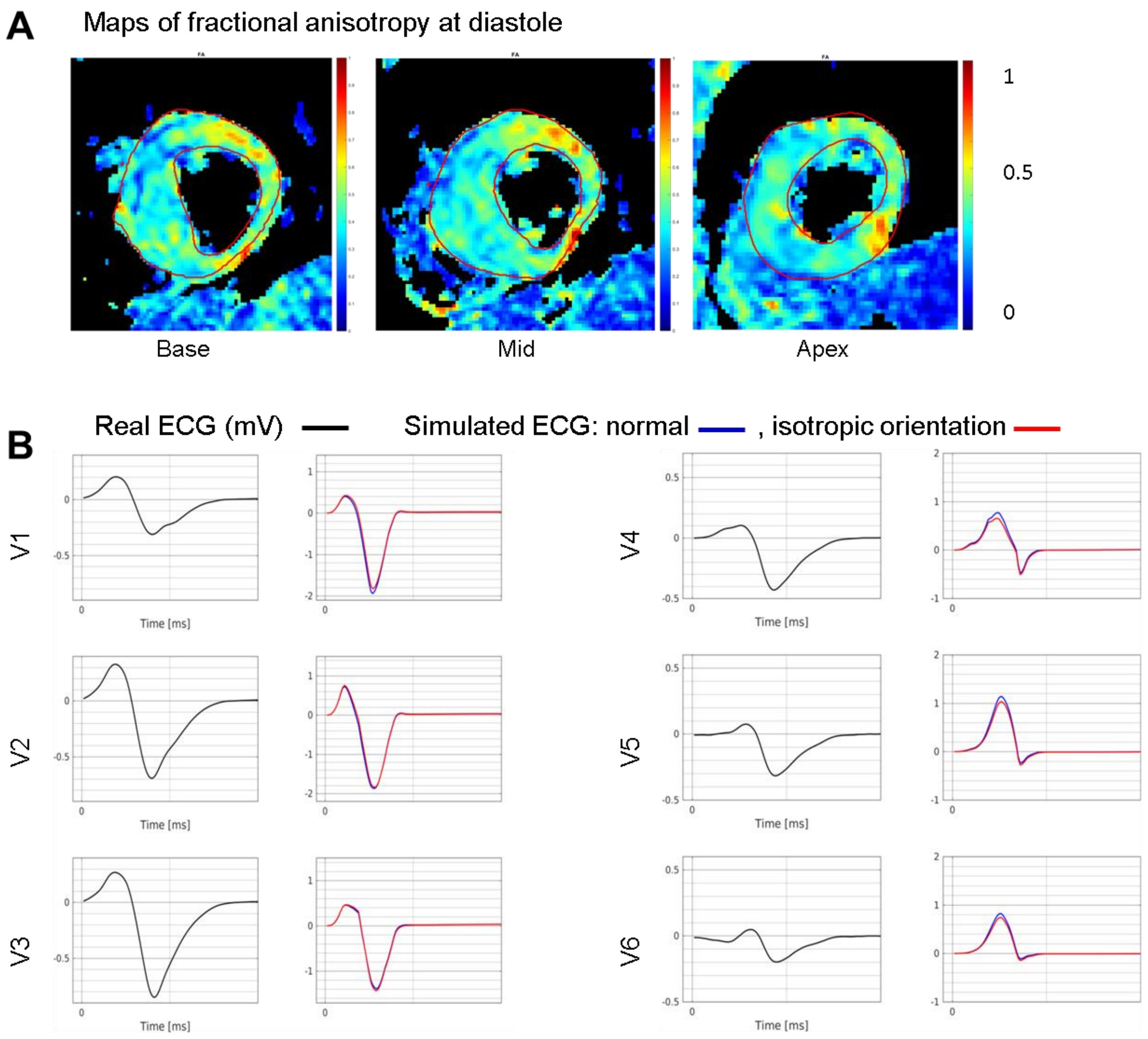

Figure S3. (Panel A) Fractional anisotropy maps for Group C patient, at diastole, for basal, mid and apical slices. Red contours delimit endocardium and epicardium. Blue regions in the myocardium correspond to areas of low fractional anisotropy. (Panel B) Simulated ECG with isotropic orientation of the myocyte fibres (red) along with normal simulation (blue) and real ECG (black) in leads V1 to V6.

2.3 Effect of slow conduction in different regions of the myocardium

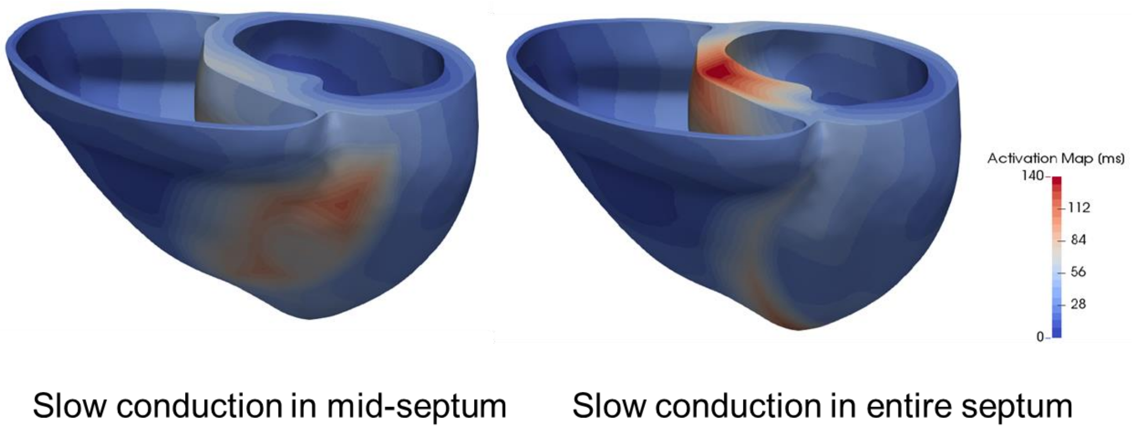

Figure S4. Activation maps (ms) in the case of slow conduction in the mid-septum (left) and slow conduction in the entire septum from base to apex (right). Large activation delays are observed in the anterior of the ventricles caused by the region of slow conduction in the mid septum, which extended to the anterior, but these are not present in the entire septum case.

Supplemental references

1. Villard B, Zacur E, Dall'Armellina E, Grau V. Correction of Slice Misalignment in Multi-breath-hold Cardiac MRI Scans. In: Mansi T, McLeod K, Pop M, Rhode K, Sermesant M, Young A, editors. Statistical Atlases and Computational Models of the Heart Imaging and Modelling Challenges. Springer International Publishing; 2017. p. 30–8.

2. Villard B, Grau V, Zacur E. Surface Mesh Reconstruction from Cardiac MRI Contours. J Imaging. 2018 Jan 10;4(1):16.

3. Zacur E, Minchole A, Villard B, Carapella V, Ariga R, Rodriguez B, et al. MRI-Based Heart and Torso Personalization for Computer Modeling and Simulation of Cardiac Electrophysiology. In: Cardoso MJ, Arbel T, Tavares JMRS, Aylward S, Li S, Boctor E, et al., editors. Imaging for Patient-Customized Simulations and Systems for Point-of-Care Ultrasound. Springer International Publishing; 2017. p. 61–70.

4. Si H. TetGen, a Delaunay-Based Quality Tetrahedral Mesh Generator. ACM Trans Math Softw. 2015 Feb;41(2):11:1–11:36.

5. Cardone-Noott L, Bueno-Orovio A, Mincholé A, Zemzemi N, Rodriguez B. Human ventricular activation sequence and the simulation of the electrocardiographic QRS

complex and its variability in healthy and intraventricular block conditions. Europace. 2016 Dec;18(Suppl 4):iv4–15.

6. Niederer SA, Kerfoot E, Benson AP, Bernabeu MO, Bernus O, Bradley C, et al. Verification of cardiac tissue electrophysiology simulators using an N-version benchmark. Philos Transact A Math Phys Eng Sci. 2011 Nov 13;369(1954):4331–51.

7. O’Hara T, Virág L, Varró A, Rudy Y. Simulation of the undiseased human cardiac ventricular action potential: model formulation and experimental validation. PLoS Comput Biol. 2011 May;7(5):e1002061.

8. Dutta S, Mincholé A, Quinn TA, Rodriguez B. Electrophysiological properties of computational human ventricular cell action potential models under acute ischemic conditions. Prog Biophys Mol Biol. 2017 Oct;129:40–52.

9. Caldwell BJ, Trew ML, Sands GB, Hooks DA, LeGrice IJ, Smaill BH. Three distinct directions of intramural activation reveal nonuniform side-to-side electrical coupling of ventricular myocytes. Circ Arrhythm Electrophysiol. 2009 Aug;2(4):433–40.

10. Clerc L. Directional differences of impulse spread in trabecular muscle from mammalian heart. J Physiol. 1976 Feb;255(2):335–46.

11. Gabriel S, Lau RW, Gabriel C. The dielectric properties of biological tissues: III. Parametric models for the dielectric spectrum of tissues. Phys Med Biol. 1996 Nov;41(11):2271–93.

12. Rush S, Abildskov JA, Mcfee R. Resistivity of Body Tissues at Low Frequencies. Circ Res. 1963 Jan 1;12(1):40–50.

13. Dijkstra EW. A Note on Two Problems in Connexion with Graphs. Numer Math. 1959;1(1):269–271.

14. Streeter DD, Spotnitz HM, Patel DP, Ross J, Sonnenblick EH. Fiber orientation in the canine left ventricle during diastole and systole. Circ Res. 1969 Mar;24(3):339–47.

15. Pitt-Francis J, Pathmanathan P, Bernabeu MO, Bordas R, Cooper J, Fletcher AG, et al. Chaste: A test-driven approach to software development for biological modelling. Comput Phys Commun. 2009 Dec 1;180(12):2452–71.
